# THERE ARE NO ADVANTAGES BETWEEN LAPAROSCOPIC AND OPEN LIVER RESECTIONS WITHIN AN ENHANCED RECOVERY PROGRAM (ERAS)

**DOI:** 10.1590/0102-672020210002e1593

**Published:** 2021-10-18

**Authors:** Gustavo Adrian NARI, Ernesto Castro GUTIERREZ, Jose Luis LAYUN, Laia FALGUERAS, Daniela MARIOT, Georgina FERRET, Celia CAULA, Javier GÓNGORA

**Affiliations:** 1 Hospital Transito Caceres de Allende, Cirugía, Córdoba, Argentina; 2 Servicio de Cirugia Hepatobiliopancreatica, Hospital Josep Trueta, Girona, Espanha; 3 Instituto de Salubridad del Estado de Aguascalientes, Estadisticas, Aguascalientes, México

**Keywords:** Hepatectomy, Laparoscopy, Enhanced recovery after surgery, Hepatectomia, Laparoscopia, Recuperação pós-cirúrgica melhorada

## Abstract

**Background:**

The use of a successful Enhanced Recovery After Surgery (ERAS) in colorectal surgery favored its application in other organs, and hepatic resections were not excluded from this tendency. Some authors suggest that the laparoscopic approach is a central element to obtain better results.

**Aim:**

To compare the laparoscopic vs. open hepatic resections within an ERAS to evaluate if there are any differences between them.

**Methods:**

In a descriptive study 80 hepatic resections that were divided into two groups, regarding to whether they were submitted to laparoscopy or open surgery. Demographic data, those referring to the hepatectomy and the ERAS was analyzed.

**Results:**

Forty-seven resections were carried out in open surgery and the rest laparoscopically; in the first group there was only one conversion to open surgery. Of the total, 17 resections were major hepatectomies and in 18 simultaneous resections. There were no differences between procedures regarding hospital stay and number of complications. There was a greater adherence to the ERAS (p=0.046) and a faster ambulation (p=0.001) in the open surgery.

**Conclusion:**

The procedure, whether open or laparoscopically done in hepatic resections, does not seem to show differences in an ERAS evaluation.

## RESUMO

**Racional:**

O uso do protocolo Recuperação Otimizada Após Cirurgia (ERAS/ACERTO) com sucesso na cirurgia colorretal favoreceu a aplicação dele em outros órgãos; as ressecções hepáticas não foram excluídas dessa tendência. Alguns autores sugerem que a abordagem laparoscópica é elemento central para a obtenção de melhores resultados.

**Objetivo:**

Comparar as ressecções hepáticas laparoscópicas e abertas dentro de um ACERTO para avaliar se existem diferenças entre as duas técnicas.

**Métodos:**

Estudo descritivo comparando 80 ressecções hepáticas divididas em dois grupos, as realizadas por laparoscopia e aquelas por laparotomia. Foram analisados dados demográficos, referentes à hepatectomia e ao ACERTO.

**Resultados:**

Foram realizadas 47 ressecções por laparotomia e o restante por laparoscopia; houve apenas uma conversão para laparotomia no grupo da laparoscopia. Do total, 17 ressecções foram hepatectomias maiores e em 18 ressecções simultâneas. Não houve diferenças entre os procedimentos quanto ao tempo de internação e número de complicações. Houve maior adesão ao ACERTO (p=0,046) e deambulação mais rápida (p=0,001) na operação aberta.

**Conclusão:**

O procedimento, seja laparotômico ou laparoscópico nas ressecções hepáticas, não parece mostrar diferenças em uma avaliação ERAS/ACERTO.

## INTRODUCTION

The Enhanced Recovery After Surgery (ERAS) programs were introduced by Kehlet and Wilmore^10^ in colorectal surgery, later being used in different diseases and viscera ^4^.

The majority of authors agree that the decrease of hospital stay, costs and complications are the main advantages^8^ ^,^ ^11^ ^,^ ^13^ ^,^ ^20^ ^,^ ^23^ ^,^ ^24^ ^,^ ^25^. Hepatectomies haven’t been excluded from this trend and multiple studies, sometimes with heterogeneous programs, have been published reporting encouraging results, noticing an important decrease in hospital stay and with that a lowering of costs of US$ 2,000^3^ ^,^ ^12^ or even more^25^. Another point that some authors mention is a decrease in the number of complications, although the literature is quite contradictory in this issue^5^. In a study previously published where we compare open hepatectomies due to metastasis of colorectal origin inside and out of an ERP, we observed that there was a reduction of up to 50% in hospital stay but no difference in the percentage of complications^14^.

The minimally invasive approach is considered by some authors fundamental for the success of an ERAS^3^. Papers comparing open vs. laparoscopic liver resections within an ERAS are scarce.

The aim of this study was to compare the results between open vs. laparoscopic hepatectomies within an identical ERAS by two surgical teams. 

## METHOD

### Study design

This is a cross-sectional, observational and descriptive analysis of patients treated by two surgical teams in three different institutions on the application of an ERAS comparing the results of open vs. laparoscopic hepatectomies in three years. The laparoscopic hepatectomies were carried out in their totality by the surgical team of Hospital Josep Trueta, Girona, Espanha, while the majority of the open hepatectomies were carried out in the other two institutions.

### Enhanced Recovery After Surgery (ERAS) program

An ERAS with basic points with a higher level of evidence recommended by the ERAS society was agreed by both teams and applied to all patients in the study with previously signed informed consent.

#### Preoperative

Complete information about the procedure, advantages and full scope of it, actively involving the patient in their development.

#### Preoperative day

Diet night before the surgery: fluid intake of liquids high in carbohydrates up to 2 h prior surgery; use of low molecular weight heparin 12 h before surgery.

#### Intraoperative

Fluid therapy control (near zero balance); restriction where possible use of drains by choice of each surgeon; nasogastric intubation with withdrawn at the end of surgery; epidural catheter or intrathecal morphine.

#### Postoperative

Withdrawal of fluid therapy within the first 48 h; regulated use of prokinetics; start oral liquid intake within the first 24 h; start of a regular diet within 48 h; bladder catheter removal within the first 24 h; early beginning of ambulation.

#### Criteria to consider total functional recovery

Pain control with oral analgesics; no use of intravenous fluids; mobilization by their own means at the preoperative level or similar; solid food intake; normal bilirubin levels or starting to decrease towards normality; total functional recovery assessed at the time of patient discharge.

The adherence to the ERAS was made through a checklist with the points of the program previously described in which each surgeon marked “yes” or “no” according to compliance with each of the items on the list. 

Demographic data was evaluated, as were those referring to etiology and hepatectomies, the postoperative course and from the data from the ERAS. The hepatectomies were classified according to the Brisbane classification; the bile leakage according to the proposal of the International Study Group of Liver Surgery; liver failure according to the 50/50%; criterion and complications according to the Dindo-Clavien classification.

Surgery with controlled decrease in central venous pressure was performed in all patients and mean arterial pressure was measured in all of them. Lower limb bandage was used in all patients as a measure of anti-thrombotic prophylaxis. Antibiotic prophylaxis was used in all patients. Intraoperative ultrasound was used in all patients to investigate lesions and to recognize intra-hepatic anatomy.

### Statistical analysis

Categorical variables were described using proportions, symmetric continuous variables using mean and standard deviation, and asymmetric continuous variables using median and range. The chi-square test was used to compare proportions between groups as well as Fisher’s exact test when appropriate. For the comparisons of average between groups, the Mann-Whitney U test was used, which was considered more appropriate than Student’s T test due to the reduced sample size. In all cases, a bilateral alpha error of <5% was considered. The data was analyzed with the PASW 18 software. 

## RESULTS

Eighty liver resections were incorporated into the study, 47 resections (58.7%) with the open approach and the rest laparoscopically; there was only one conversion (3.03%). Of the total, 17 (21.2%) were major hepatectomies (resection of three or more consecutive Couinaud liver segments) and in 18 (22.5%) simultaneous resections of liver metastases and primary colorectal cancer were performed. 85% of the resections were indicated because of malignant disease and of these the vast majority by metastases from colorectal cancer. The hospital stay of the entire series had an average of 3.9 days, adherence to the protocol was of 72.5% and full recovery of 85%. The rest of the general data of the series can be seen in Table 1. 

Table 2 shows the demographic data of both groups where no differences were observed regarding age, gender or BMI, highlighting a greater number of patients with lower surgical risk in patients undergoing open surgery and a higher alcohol consumption in the group of patients who underwent laparoscopic surgery. 

Table 3 shows the data regarding hepatectomies. Although there was no statistical significance, in the open surgery group there were a greater number of major liver resections (12-25.5% vs. 4-15.1%) and simultaneous resections of both the liver metastases and the primary colorectal tumor (14-29.7% vs. 1-12.1%).

**TABLE 1:** General data of the series in 80 liver resections

Variable	Number % Median rank
Age	59.28 years (28-84)
Male	47 (53.75%)
BMI	26.76 (18-46)
Major hepatectomies	17 (21.25%)
Open way	47 (58.75%)
Simultaneous resection	18 (22.5%)
Repeated resection	10 (12.5%)
Operative time in minutes	204 min (20-530)
Hospital stay	3.9 days (2-9)
Hospital re-entry	4 (5%)
Re-operation	1 (1.25%)
Adherence	58 (72.5%)
Full recovery	68 (85%)
Indication	80
MTS CCR	48
HCC	8
MTS breast	4
Other MTS	8
Hydatidosis	5
Hemangioma	3
FNH	2
Others	2
Complications	13 (16.25%)

MTS CCR=metastasis from colorectal cancer; HCC=hepatocellular carcinoma; MTS=metastasis; FNH=focal nodular hyperplasia

**TABLE 2:** Demographic data

Variable	Open surgery (n=47)	Laparoscopic surgery (n=33)	p
Male	51.06%	57.57%	0.565
Median age - SD (years)	57+/- 14	61 +/- 17.5	0.090
ASA2	44.68%	15.15%	0.013
ASA3	55.31%	81.81%	
ASA4	-	3.03%	
Alcohol comsumption	4.25%	21.21%	0,018
Tabaco	19.14%	30.30%	0,248
BMI (kg/m^2^)	26.24	27.27	0.742
Physical excercise	29.78%	51.51%	0.050

**TABLE 3:** Data of liver resections

Variable	Open surgery (n=47)	Laparoscopic surgery (n=35)	p
Major hepatectomy	25.53% (12)	15.15% (5)	0.354
Simultaneous resection	29.78% (14)	12.12%(4)	0.062
Operative time (min)	155	232.62	0.001
Blood loss >300 ml	46.80%	57.57%	0.393
Complications Dindo Clavien	14.89% (7) I/5 II/1 V/1	18.18% (6) I/1 II/3 IIIA/2	0.695
Use of drains	46.8% (22)	21.21% (7)	0.019
Hospital stay without re-entry (days)	4	3	0.5323
Hospital stay with re-entry (days)	5.25	7	0.235

In both groups there were no differences in terms of complications (n=7, 14.8% vs. n=6, 18.1%), in the open surgery group there were five complications type I and one complication type 5 that represents the only cause of death of the series, while in the laparoscopic approach there were more complications type II and III. The hospital stay did not present significant differences between both groups, both with and without readmission, the average number of days in the laparoscopic surgery group when readmissions were counted amounted to seven days, and this was partly due to the fact that complications in this group were more serious. There was a significant difference (p=0.019) regarding the placement of drains in the abdominal cavity; they were used 22 times (46.8%) in open surgery and seven (21.2%) in laparoscopic surgery. The greater number of simultaneous and major resections in open surgery group conditioned this difference. On the other hand, the operative time was shorter in open surgery than when the laparoscopic approach was performed (p=0.001)

There was a single death due to respiratory failure in the immediate postoperative period after a right trisectionomy (2.12%), while in the laparoscopic approach there was no mortality. 

**TABLE 4:** ERAS results

Variable	Open surgery (n=47)	Laparoscopic surgery (n=33)	p
Adherence	80.85%	60.60%	0.046
Start of ambulation	16 (RI:12-24)	24 (RI:23-36)	0.001
Bladder catheter removal (hours)	18	24	0.022
Amdominal drains removal (hours)	38.04	80	0.122
Intravenous fluis removal (hours)	36	30	0.255
Full recovery	80.85% (38)	90.90% (30)	0.215
Hospital re-entry	2 (2.5%)	2 (2.5%)	0.230

Table 4 shows the results of ERAS implemented, where the outstanding data and the most significant difference observable are due to a greater adherence to the program (p=0,046), the start of ambulation (p=0.001) and the withdrawal of the bladder catheter (p=0.022) that point in favor of open surgery. Meanwhile, although there was no significant difference in terms of full recovery, better performance was observed in the laparoscopic surgery group.

## DISCUSSION

The success of the application of an ERAS in major colon and rectal surgery has encouraged its application in other organs. The use of an ERAS in liver surgery has been increasingly indicated in different surgery services. It is known that the application of an ERAS has been beneficial due to the decrease in hospital stay and therefore costs^17^ ^,^ ^22^. Only few studies compared the results of these programs in open vs. laparoscopic hepatectomies. The minimally invasive approach has been considered to be one of the main points in regards to obtaining a faster and better quality of functional recovery when compared to the open approach in patients undergoing liver resections ^16,18,19^. These studies indicate a great improvement in terms of hospital stay, decrease in complications and less blood loss with the consequent decrease in transfusion needs when compared to liver resections with an open approach, with the addition that the laparoscopic approach would decrease the postoperative ileus time. Other authors assure that the laparoscopic approach favors an earlier start of oral intake and a lesser need for intravenous analgesics; all these studies were performed outside an ERAS ^16,18,19^ and our study mainly aimed to compare both approaches within one. 

For this study, an ERAS was agreed between two surgical teams, taking into account the points with the highest level of evidence recommended and suggested by the ERAS Society. 

From the analysis of the results and attending to the primary objectives of our work, we noted that the hospital stay did not show significant differences between the two groups. Most series report a hospital stay of between 3-7 days^47,53^ confirming a decrease of up to 50% in hospital days when compared to conventional management outside an ERAS.

The decrease in hospital stay produces a significant reduction in costs^24^ ^,46^. However, there were patients who underwent different simultaneous resections which could have prolonged the hospital stay. This did not happen when we consider the results reported in other studies^26^ ^,^ ^30^.

When we compare the complications between the open and laparoscopic approach, there were no differences either, this percentage of complications is similar to a previous publication^15^. In the open surgery group, there were a greater number of Dindo-Clavien type I complications and they were more related to complications of the surgical wound and a serious complication that triggered the only death in the series. On the laparoscopic approach, there were a greater number of type II and III complications, which favored a greater number of days in stay during the readmissions in this group. At this point, one might wonder if a greater use of drains in laparoscopic surgery would not have significantly reduced the number of complications and therefore hospital stay. The percentage of complications reported in other series is around 30%^26^, a probable explanation for a low number of complications in our groups would be a high number of minor liver resections, this would also coincide with some authors who noted different results when stay and morbidity were compared between minor and major hepatectomies; on the other hand, some authors report a percentage similar to our in laparoscopic resections, although the number of minor hepatectomies in these series was predominant, reaching 91% in Savikko’s series ^22^.

Adherence to the ERAS showed a difference in favor of open surgery (p=0.046), in the same way as the start of ambulation (p=0.001). A possible explanation for this difference in the start of ambulation may lie in two points: on the one hand, that laparoscopic surgery requires the use of pneumoperitoneum, which may favor the appearance of omalgia and other disorders that would delay the start of mobilization; and on the other, that when the surgical times were compared, there were differences in favor of the open technique (p=0.001), it is known that longer anesthetic and surgical times have an impact on recovery time and therefore on the start of ambulation and the decrease in complexity of the patients. Some authors report very detailed information on what is expected of patients in the postoperative period, a multidisciplinary approach and a staggered start of the mobilization of the patient indicating that they remain seated in bed or in a chair in the first 24 h to later progress to ambulation favors a greater adherence^27^. At this point we fully agree that information to the patient and the family is essential to receiving collaboration in order to achieve greater adherence. 

Blood loss is also a factor that can affect postoperative adherence, playing an important role in the decrease of the complexity in patients, different authors have stated that the laparoscopic approach has a lower rate of bleeding when compared with open approaches in the comparison of both groups there were no differences regarding blood loss^30^. 

Full recovery at discharge did not show significant differences either (p=0.215) although in laparoscopic surgery there was a higher percentage of patients who reached this state. We noted in open surgery that postoperative pain control at discharge was less satisfactory. It is likely that, as well as the use of pneumoperitoneum and a longer operative time could negatively affect the start of ambulation, minimal invasive procedures achieve better control of postoperative pain at discharge favoring greater total recovery. Another point that we analyzed and that although it did not present a significant difference but could play an important role in pain control is that in the open surgery group there were a greater number of simultaneous resections. Wong Lun Hing et al^28^ achieved full recovery in the majority of their patients on the 5^th^ day of the postoperative period, which coincides with our results.

Other data that was compared was the use of cavity drains, which was greater in open surgery, an explanation for this difference lies in a greater number of simultaneous resections. In the open surgery group, drainage was placed in 46.8%, some series report the use of drains in around 20% with the open technique^3^. We agree with the authors who report that the use of drains is unnecessary in a significant number of patients^29^; for this purpose, it would be correct to protocolize in which patients they should be placed. Some authors suggest that the placement of drains in hepatectomies should be performed in patients whose surgery lasts more than 350 min, a blood loss of more than 650 ml or with bile leak or doubt regarding it during surgery. 

We can express that our study presents several weaknesses: there was no randomized distribution of patients, the number of patients should be greater, and laparoscopically operated patients have been operated by a single center. On the other hand, an ERAS in liver surgery brings benefits to patients in terms of hospital stay in coincidence with other authors. Perhaps a greater number of cases, the division between major and minor hepatectomies and the exclusion of simultaneous resections would allow us to produce more certain result.

## CONCLUSION

The procedure, whether open or laparoscopically, done in hepatic resections, does not seem to show differences in an ERAS evaluation.
